# Tissue Inhibitor Of Matrix Metalloproteinase-1 Is Required for High-Fat Diet-Induced Glucose Intolerance and Hepatic Steatosis in Mice

**DOI:** 10.1371/journal.pone.0132910

**Published:** 2015-07-13

**Authors:** Even Fjære, Charlotte Andersen, Lene Secher Myrmel, Rasmus Koefoed Petersen, Jakob Bondo Hansen, Hanne Sørup Tastesen, Thomas Mandrup-Poulsen, Nils Brünner, Karsten Kristiansen, Lise Madsen, Maria Unni Rømer

**Affiliations:** 1 Department of Biology, Faculty of Science, University of Copenhagen, Copenhagen, Denmark; 2 National Institute of Nutrition and Seafood Research, Bergen, Norway; 3 Department of Veterinary Disease Biology, Faculty of Health and Medical Sciences, University of Copenhagen, Frederiksberg, Denmark; 4 Department of Biomedical Sciences, Faculty of Health and Medical Sciences, Copenhagen, University of Copenhagen, Denmark; 5 Department of Molecular Medicine and Surgery, Karolinska Institutet, Stockholm, Sweden; 6 Department of Clinical Physiology, Nuclear Medicine and PET, Rigshospitalet, University of Copenhagen, Copenhagen, Denmark; University of Texas Health Science Center at Houston, UNITED STATES

## Abstract

**Background:**

Plasma levels of tissue inhibitor of matrix metalloproteinase-1 (TIMP-1) are elevated in obesity and obesity-related disorders, such as steatosis, but the metabolic role of TIMP-1 is unclear. Here we investigated how the presence or absence of TIMP-1 affected the development of diet-induced glucose intolerance and hepatic steatosis using the *Timp1* null mice.

**Methods:**

*Timp1* knockout (TKO) and wild type (TWT) mice were fed chow, high-fat diet (HFD) or intermediate fat and sucrose diet (IFSD). We determined body weight, body composition, lipid content of the liver, energy intake, energy expenditure, oral glucose tolerance, as well as insulin tolerance. In addition, the histology of liver and adipose tissues was examined and expression of selected genes involved in lipid metabolism and inflammation in liver and adipose tissues was determined by RT-qPCR.

**Results:**

TKO mice gained less weight and had lower energy efficiency than TWT mice when fed HFD, but not when fed chow or IFSD. Importantly, TKO mice were protected from development of HFD- as well as IFSD-induced glucose intolerance, hepatic steatosis, and altered expression of genes involved in hepatic lipid metabolism and inflammation.

**Conclusion:**

Collectively, our results indicate that TIMP-1 contributes to the development of diet-induced hepatic steatosis and glucose intolerance and may be a potential therapeutic target.

## Introduction

Obesity is associated with low-grade inflammation and increases the risk of chronic diseases such as type 2 diabetes, coronary heart disease, hypertension, dyslipidemia and non-alcoholic steatohepatitis (NASH) predisposing to premature death. Extracellular matrix remodeling by the balanced action of metalloproteinases (MMP) and MMP inhibitors is central not only for tissue structure but also function and metabolism. Recent interest has focused on the role of the extracellular matrix in adipogenesis and insulin resistance, as genetic ablation of MMP3, 11 and 19 enhances diet-induced obesity in mice [1–4]. In humans, tissue inhibitor of matrix metalloproteinase-1 (TIMP-1) has been described as a biomarker of obesity [5–7], dyslipidemia [8–10], NASH [11–13], cardio-vascular disease [14] and type 2 diabetes [15]. TIMP-1 deficiency has been reported to increase body weight (BW) and fat mass in female mice fed a low fat chow diet [16]. This was suggested to be caused by hyperphagia elicited via hypothalamic pathways and reduced thermogenesis, but peripheral actions promoting adipogenesis may also contribute. However, this may be gender specific, since male TIMP-1 deficient mice fed a high-fat diet (HFD) were reported to have decreased BW and fat mass [17].

The role of TIMP-1 in liver metabolism, insulin resistance and inflammation remains to be elucidated. Hepatic *Timp1* expression is increased by euglycemic hyperinsulinemia in both insulin-sensitive and insulin resistant rats [18]. Hepatic *Timp1* expression is also elevated in a mouse model that resembles type 2 diabetes, elicited by HFD combined with low doses of strepzotocin treatment in order to induce beta cell dysfunction. [19]. Furthermore, Meissburger *et al*. [20] found that administration of recombinant TIMP-1 to male mice fed a HFD increased the levels of circulating non-esterified fatty acids, enhanced hepatic triacylglycerol accumulation, and accelerated insulin resistance. This suggests that TIMP-1 might promote accumulation of hepatic triglycerides and hepatic insulin resistance. As data still is limited, we examined diet-induced obesity, hepatic steatosis and insulin resistance in *Timp1* null mice, bred on a BALB/c background. As BALB/c mice have an intermediate response to diet-induced diabetes [21], the mice were fed an intermediate fat and sucrose diet (IFSD), or a HFD for 26–29 weeks in order to mimic age-induced type 2 diabetes better in this mouse strain than in a rapidly (6–8 weeks induction) inducible mouse strain like the C57BL/6J mouse.

## Materials and Methods

### Ethics statement

The animal studies were conducted in accordance to NIH principles of laboratory animal care. The protocol for the studies was approved by The Danish Animal Experimental Board (permit number 2008/561-1536).

### Animals

*Timp1* knockout (TKO) [22] and wild type (TWT) mice were bred at Taconic Europe (Denmark) and TKO and TWT mice were backcrossed onto BALB/c background for ten generations as previously described [23]. Animals were housed in a 12-h light/dark cycle at 22°C. The mice used in this study were bred on a BALB/c background, and genotyping was performed as previously described [23]. Wistar rats used for pancreas islet isolation were obtained from Taconic Europe (Denmark).

Dietary allocation: Seventeen week-old male TKO or TWT mice were randomized to either chow (Altromin 1319, Brogaarden, CE Denmark) or HFD (D12492, Research Diets Inc., New Brunswick, NJ, USA) and fed *ad libitum* for 26 weeks (n = 11 per group). Fourteen week-old male TKO and TWT mice were fed IFSD (Ssniff Spezialdiäten, Soest Germany) *ad libitum* for 29 weeks (n = 10 per group). The compositions of the diets are summarized in S1 Table.

Body weight (BW) and feed intake were recorded weekly. Mice were killed by cervical dislocation. Heart blood was immediately drawn into heparin-coated tubes (Sigma-Aldrich, Brøndby, Denmark) and plasma was collected after centrifugation. White adipose tissues (WAT), including inguinal WAT (iWAT), epididymal WAT (eWAT), perirenal WAT (pWAT), in addition to interscapular brown adipose tissue (iBAT) and liver were dissected out. Half of the biopsies were freeze-clamped in liquid nitrogen and kept at -80°C and the other half fixed in 4% phosphate buffered paraformaldehyde, dehydrated and embedded in paraffin. The tibialis anterior muscle was dissected out, freeze-clamped and kept at -80°C. Whole pancreas was dissected and placed in acid ethanol for insulin extraction.

### Indirect calorimetry

Oxygen consumption, CO_2_ production, and heat production were measured for 24 h using computerized metabolic cages (Labmaster system, TSE Systems, Bad Homburg, Germany). The mice were acclimatized to the metabolic cage environment one day prior to start of the monitoring period.

### Glucose and insulin tolerance tests

Oral glucose tolerance test (OGTT): The mice were fasted for 16 h (overnight) and gavaged 2 g glucose/kg BW.

Insulin tolerance test (ITT): The mice were fasted for 6 h and injected intraperitoneally (i.p.) with 0.75 U/kg BW of human insulin (Humulin R, Eli Lilly, Herlev, Denmark). All animals had free access to water during the fasting and all experimental tests. In both tests blood glucose was measured in tail vein blood at indicated time points with a Contour glucometer (Bayer, Copenhagen, Denmark).

Insulin secretion: The mice were fasted for 4 h and gavaged 2 g glucose/kg BW. Blood samples were collected from the retro-orbital sinus into EDTA-coated tubes (Sarstedt, Nümbrecht, Germany). Plasma was obtained by centrifugation and stored at -80°C until analysis. Plasma insulin was quantified with ELISA at Novo Nordisk (Måløv, Denmark) using the "Ultrasensitive rat insulin ELISA kit" (Crystal Chem, Downers Grove, IL, USA) with the following modifications: Sample volume was reduced to 5 μl, and kit standards were replaced with in-house rat insulin standards prepared using heat treated rat plasma. The lower limit of quantification was 50 pM.

### Body composition scanning

Body composition was determined by quantitative magnetic resonance scanning using EchoMRI 2000 4-in-1 mouse Composition Analyzer (Echo Medical Systems, Houston, TX, USA).

### Residual fecal energy content

After 19 weeks on the experimental diets, feces were collected over two days and the energy content was determined in an IKA Calorimeter C 5000 Control (IKA-Werke GmbH & Co. KG, Staufen, Germany).

### Pancreatic insulin quantification

Isolated pancreas was placed at -20°C overnight in ethanol-HCl (70%; 1M) and thereafter homogenized with fine scissors and extracted for further 4–5 d at -20°C. Insulin concentration was determined by insulin ELISA [24]. For protein quantification, homogenized tissue was isolated and lysed with cell lysis buffer (Cell Signaling, Glostrup, Denmark) and quantified using Pierce BCA protein quantification kit (Thermo Scientific, Hvidovre, Denmark).

### Real-time qPCR

Tissues: Total RNA was purified from liver, eWAT and iWAT from non-fasted mice at the termination of the study (n = 8–11) using Trizol (Life Technologies, Nærum, Denmark). RNA concentrations were measured on a Nanodrop (Thermo Scientific). cDNA was synthesized with RevertAid (Thermo Scientific) and stored at −80°C until analysis on LightCycler 480 (Roche, Hvidovre, Denmark). cDNA was analyzed in duplicates in 20 μl reactions containing SYBR Green JumpStart Taq ReadyMix (Sigma-Aldrich). Primers (S2 Table) were purchased from Tag Copenhagen (Frederiksberg, Denmark). Data was analyzed using Roche Lightcycler software and the ΔΔCt method, and normalized to 18sRNA or general transcription factor II B (*gtf2b*).

Islets and insulinoma cells: After termination of the experiments, INS-1 cells or islets were snap-frozen, RNA isolated using RNeasy (Qiagen, Copenhagen, Denmark), cDNA synthesized with TaqMan reverse transcription reagent, and quantitative RT-qPCR performed using the TaqMan assay on ABI 7900 HT from Applied Biosystems (Life Technologies). TaqMan primers were from Applied Biosystems.

### Histology

Liver and adipose tissues were fixed in 4% phosphate buffered paraformaldehyde and paraffin-embedded. Three μm sections from 5 mice in each experimental group were stained with Haematoxylin and Eosin (H&E). One representative micrograph of each group is shown, and three sections of adipose tissue depots from each mouse were used for quantification of mean cell diameter. Picrosirius red staining kit (ab150681) was performed on paraffin sections as described by the manufacturer (Abcam, Cambridge, UK) (n = 5).

### Triglyceride measurements

Total lipids were extracted from liver and muscle using a modified version of the Bligh and Dyer protocol. Briefly, 25 mg of tissue were homogenized in potassium phosphate buffer, and lipids extracted with chloroform: methanol (1:2). HCl was added and the chloroform phase transferred to new tubes and evaporated under nitrogen. The extract was dissolved in LPL buffer (28.75 mM PIPES, 57.41 mM MgCl2·6H2O, 0.569 mg/ml BSA-FFA, 0.1% SDS) and analyzed with a triglyceride kit (Zen-Bio, Durham, NC, USA).

### Liver enzymes

Plasma levels of alanine transaminase (ALT) and aspartate transaminase (AST) were analyzed using Biovision kits (AH Diagnostics, Aarhus V, Denmark).

### Plasma measurements

Beta-hydroxybutyrate assay kit (Abcam, Cambridge, UK) and plasma glucose assay kit (BioVision Incorporated, CA, USA) were used and the analyses performed as described by the manufacturer.

### Islet isolation

Mouse and Wistar rat islets were isolated by collagenase (Sigma-Aldrich) digestion of the pancreas. Digested pancreatic tissue was washed in HBSS containing FBS and islets were handpicked [25].

### Apoptosis measurement

Twenty-five size-matched islets from 12 week-old male TKO or TWT mice in duplicates were seeded in a 48-well dish, pre-incubated for 2 h to reduce handling stress and exposed to cytokines as described. Apoptosis was determined by Cell Death Detection ELISAPLUS (Roche).

### Western blot

Neonatal rat islets were retrieved and lysed with cell lysis buffer (Cell Signaling). Protein was quantified using the Bradford colorimeter with optical density measured at 595 nm. Recombinant rat TIMP-1 (R&D Systems, Denmark) was used as control. Anti-rat TIMP-1 antibody was from R&D Systems and antibody against tubulin was from Cell Signaling (Beverly, MA, USA).

### Statistics

All results are shown as mean ± SEM. Dixons`s Q-test was used to screen for outliers and statistical analyses of gene expression and physiological data were performed with GraphPad Prism v6.0 (GraphPad Software, Inc.). One-way ANOVA was used to compare differences between the experimental groups, followed by Fisher`s Least Square Differences test in the first experiment, and in the second experiment a regular t-test was performed to evaluate differences between the two experimental groups. Data was considered statistically significant when p < 0.05 for all tests, and different lowercase letters denote statistically different groups. Statistical tendencies 0.075 ≥ p ≥ 0.05 are shown in the figures.

## Results

### TIMP-1 deletion reduces weight gain in mice on HFD

**Fig 1:**
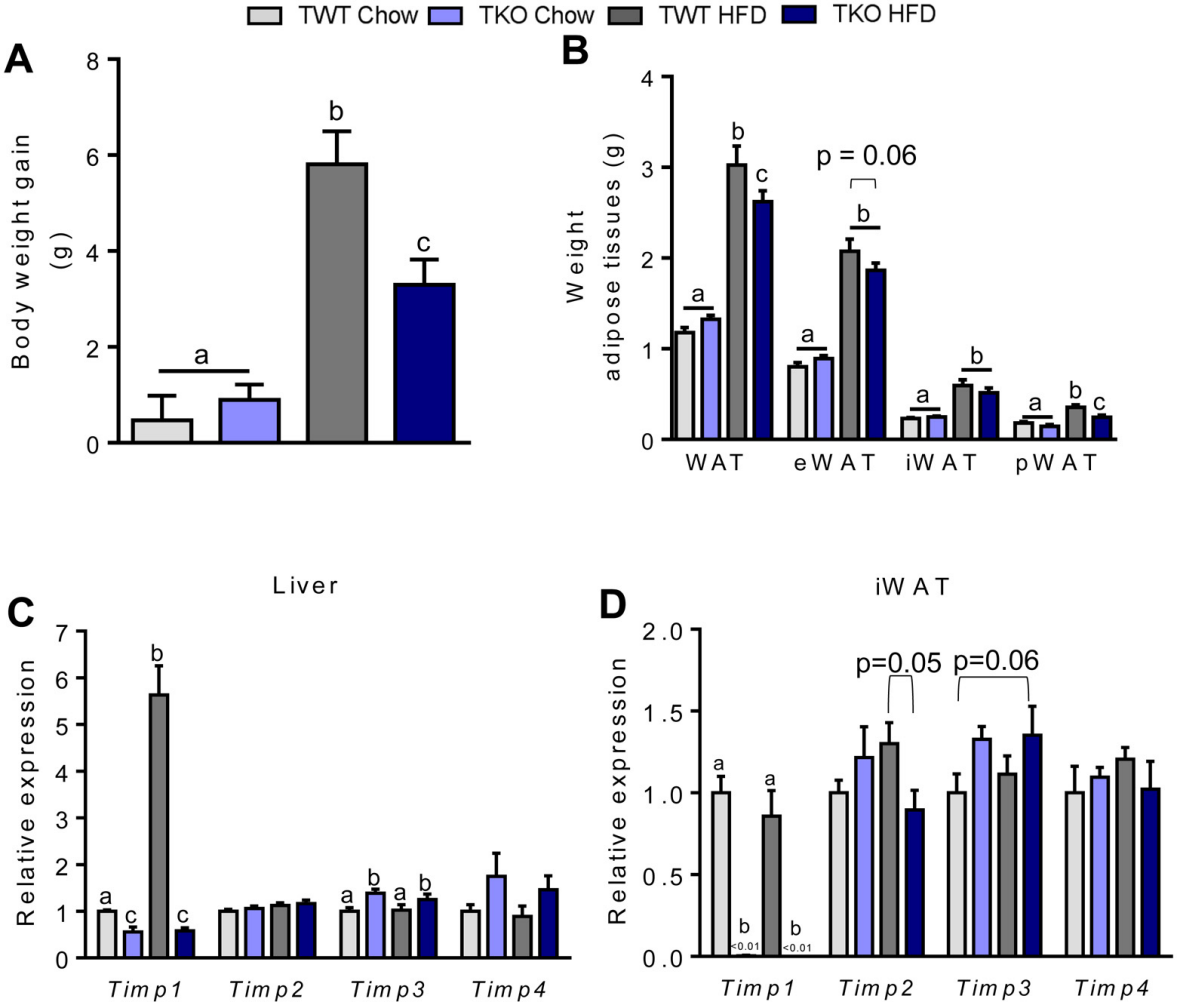
Body weight gain, adipose tissue weight and *Timp (1–4)* gene expression in wild type (TWT) and *Timp1* knockout (TKO) mice fed chow or a high-fat diet (HFD) for 26 weeks. (A) Total increase in body weight (n = 9–11). (B) Weight of white adipose tissue depots at the end of the study (n = 9–11), epididymal white adipose tissue (eWAT), inguinal white adipose tissue (iWAT), and perirenal white adipose tissue (pWAT). (C-D) Gene expression of *Timp1*, *Timp2*, *Timp3* and *Timp4* in liver and adipose tissue at the end of the study. Data was normalized to 18S ribosomal RNA or general transcription factor II B (*gtf2b*) and presented relative to the expression in TWT Chow (n = 7–10). All RT-qPCR measurements were performed in non-fasted mice. Graphs show mean ± SEM, and different lowercase letters denote statistically different groups (p < 0.05).

To elucidate the role of TIMP-1 in diet-induced development of obesity, glucose intolerance and insulin resistance we used *Timp1* knockout (TKO) mice on a BALB/c background. The TIMP-1 deletion was verified by measuring TIMP-1 levels in plasma. The plasma levels of TIMP-1 were 3717 ± 853 pg/ml in the wild type (TWT) mice, and as expected not detectable in TKO mice (data not shown). HFD feeding for 26 weeks increased body weight (BW) in both TWT and TKO compared to chow fed animals (Fig 1A). The total BW gain at the termination of the study was significantly lower in TKO than in TWT mice fed HFD. In contrast, BW gain did not differ between the genotypes when mice were fed a chow diet (Fig 1A).

To determine if the lower BW of the TKO mice after 26 weeks on HFD could be explained by differences in body composition, quantitative magnetic resonance scanning was performed. The total fat mass tended to be lower in TKO compared to TWT mice fed HFD (p = 0.06) (S1A Fig). TKO mice had significantly less white adipose tissue (WAT) compared to HFD fed TWT. However, of the different depots only the mass of the perirenal white adipose depot (pWAT) was significantly reduced. The weights of the other fat depots were comparable independent of the genotype (Fig 1B). No significant differences in total WAT mass or any specific adipose depots were observed between the genotypes, when the mice were fed a chow diet (Fig 1B).

Obesity is associated with low-grade inflammation and macrophage infiltration in adipose tissue [26]. To examine if the lower BW gain and reduced WAT mass observed in TKO-HFD mice was associated with reduced macrophage infiltration, we measured the expression of adipose tissue *Ccl2* (chemokine (C-C motif) ligand 2 also known as monocyte chemotactic protein-1). Compared with TWT mice fed chow, expression of *Ccl2* was increased in eWAT and iWAT of TWT-HFD fed mice (S1B Fig). TKO mice had a significantly lower *Ccl2* expression in eWAT compared to TWT mice, when both genotype were fed HFD, while this was not observed in iWAT. Examination and quantification of H&E stained sections of eWAT and iWAT showed significantly enlarged adipocytes in both depots of HFD-fed mice compared to chow with no difference between genotypes (S1C Fig).

### TIMP-1 deletion only slightly/marginally affects expression of Timp family members in liver and WAT

Since deletion of *Timp1* might lead to a compensatory upregulation in expression of other *Timp* family members, we measured mRNA expression levels of *Timp1*, *2*, *3* and *4* in adipose tissue and liver. A pronounced upregulation of *Timp1* mRNA expression was observed in liver of TWT mice fed HFD (Fig 1C). *Timp2* mRNA tended to be reduced (p = 0.05) in iWAT from TKO mice compared to TWT mice, when fed HFD, while *Timp3* mRNA expression was increased in liver of TKO mice when compared to the wild-type littermates irrespective of diet (Fig 1C). Apart from this, no other significant alterations in expression of *Timp* family members in liver were observed (Fig 1C). Thus, no significant compensatory effect on *Timp2* and *Timp4* expression in either liver or WAT in TKO mice were observed, while the increase in *Timp3* expression could reflect a compensatory upregulation. (Fig 1C and 1D). The mRNA expression levels of *Mmp-2* and *Mmp-9* were measured in both liver and adipose tissue. The relative hepatic expression of *Mmp-2* and *Mmp-9* was low and in iWAT the *Mmp-2* expression level was not affected by either diet or genotype. *Mmp-9* expression in iWAT was reduced in mice fed HFD compared to chow; however, this was only evident in the TWT mice and not in TKO mice (data not shown). To validate any changes in MMP-2 or MMP-9 activity zymography is necessary [27].

### Energy efficiency is decreased in TIMP-1 deficient mice

**Fig 2:**
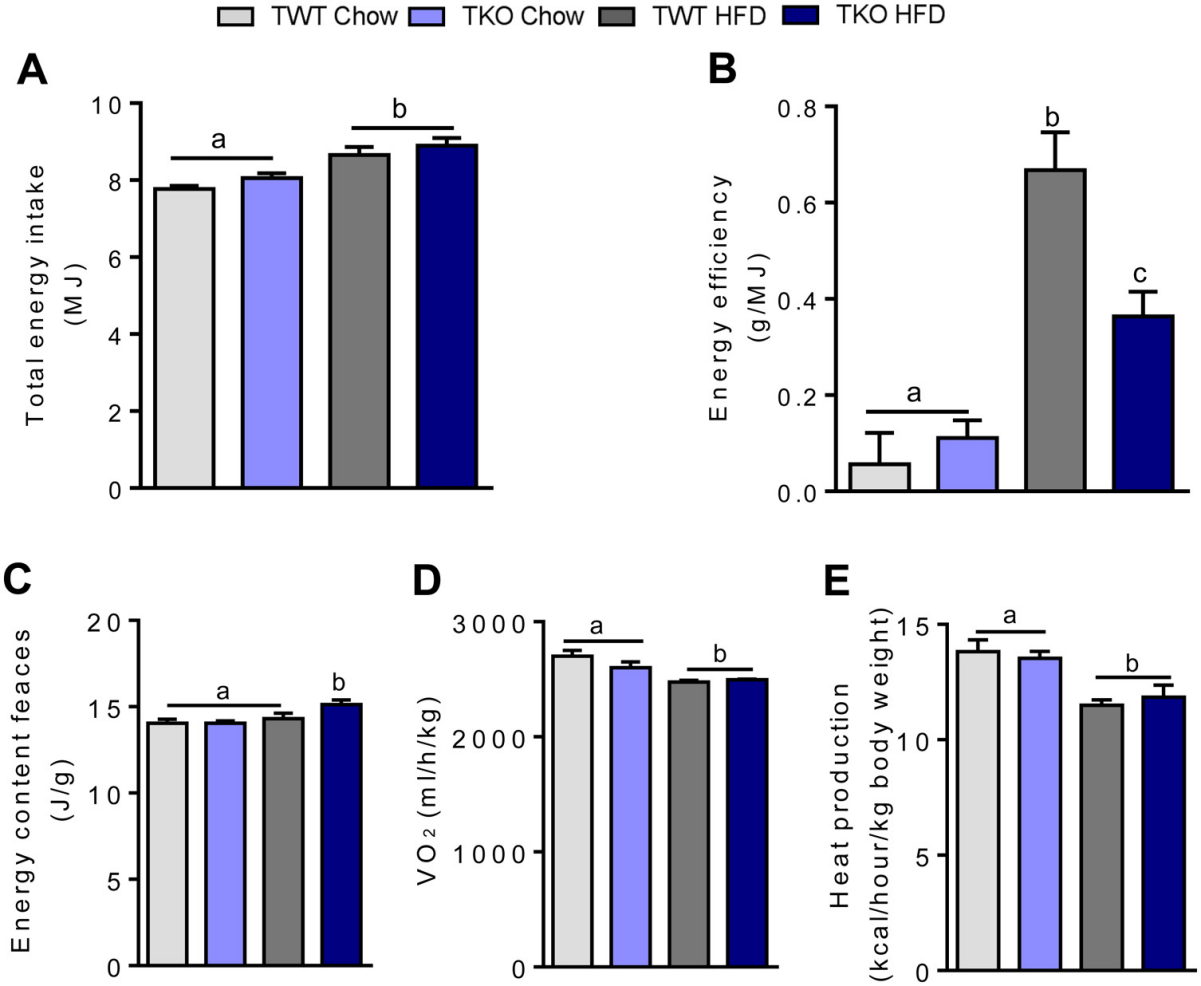
Energy uptake and metabolism in wild type (TWT) and *Timp1* knockout (TKO) mice fed chow or a high-fat diet (HFD). (A) Total energy intake (n = 9–11). (B) Energy efficiency at the end of the study measured as total body weight gain/total feed intake (n = 9–11). (C) Energy content in feces measured by calorimetry (n = 8). (D) Oxygen consumption and E heat production measured during a 24 h period with indirect calorimetry in week 20 (n = 8). Graphs show mean ± SEM, and different lowercase letters denote statistically different groups (p < 0.05).

TKO and TWT mice had equal energy intake when fed a chow or a HFD for 26 weeks; however, a significant increase in energy intake was observed in both genotypes when consuming a HFD (Fig 2A). As TKO-HFD mice had a lower BW gain, energy efficiency (defined as total body weight gain/total energy intake) was decreased by 46% in TKO-HFD compared to TWT-HFD mice (Fig 2B).

### TIMP-1 deficiency increases fecal energy loss on HFD

To assess if the reduced feed efficiency in TKO-HFD mice was due to reduced energy absorption, residual fecal caloric content was analyzed. Fecal energy content was similar for mice fed chow (Fig 2C). However, fecal energy content was significant higher (7.6%) in TKO-HFD mice compared to the TWT-HFD mice, suggesting lower energy uptake in TKO-HFD compared to TWT-HFD mice (Fig 2C).

Indirect calorimetry measurements demonstrated that oxygen consumption and heat production were lower in HFD than chow fed mice, but not influenced by the genotype in TKO-HFD and TWT-HFD mice (Fig 2D and 2E). Further, we did not detect any differences in uncoupling protein-1 (*Ucp1*) or peroxisome proliferator-activated receptor γ co-activator 1α (*Ppargc1a*) expression in the two genotypes (data not shown).

### TIMP-1 deficiency protects against diet-induced glucose intolerance

**Fig 3:**
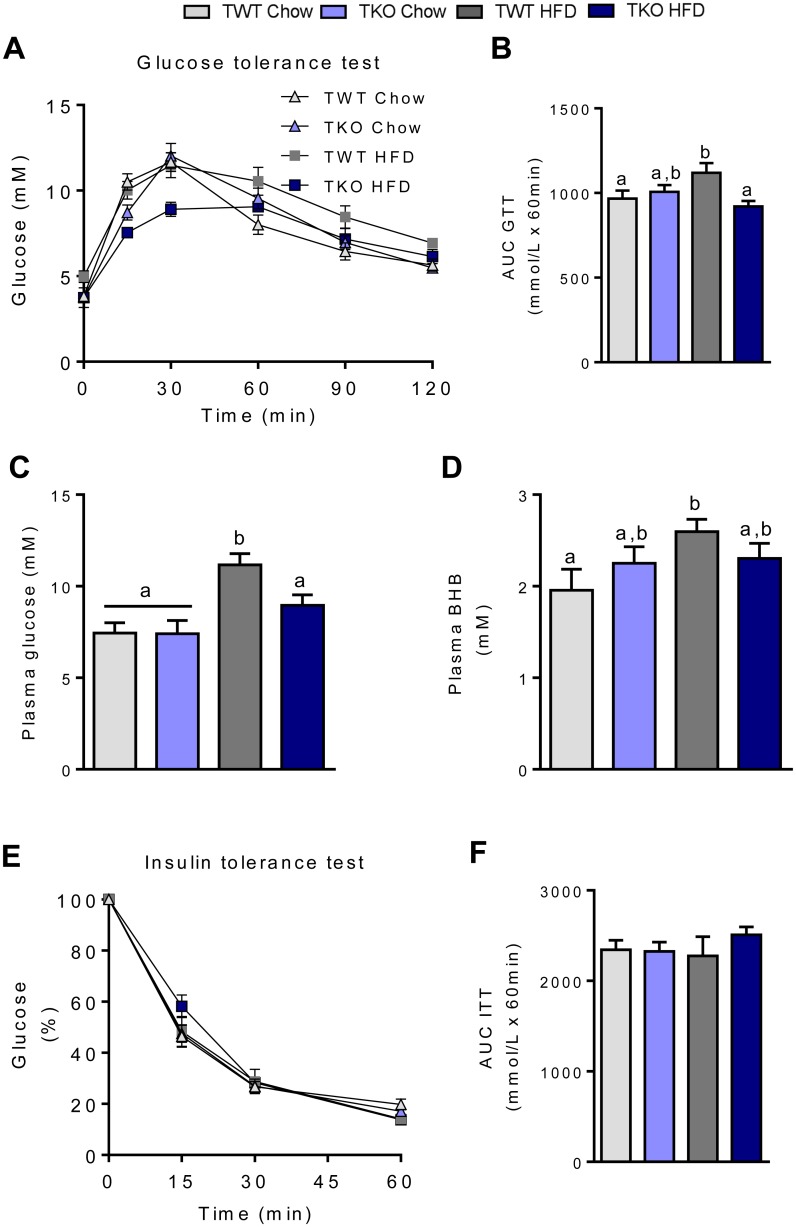
Glucose and insulin tolerance test in wild type (TWT) and *Timp1* knockout (TKO) mice fed chow or a high-fat diet (HFD). (A) Blood glucose (mM) levels after 23 weeks feeding before and after gavage of glucose (2 g/kg) (n = 8). (B) AUC values for glucose tolerance test (n = 8). (C) Plasma measurements of glucose and (D) beta hydroxybutyrate (BHB) in non-fasted mice at the termination of the study. (E) Blood glucose (mM) levels after 22 weeks feeding before and after i.p. injection of insulin (0.75 U/kg) (n = 8). (F) AUC values for insulin tolerance test (n = 8). Graphs show mean ± SEM, and different lowercase letters denote statistically different groups (p < 0.05).

To investigate if the reduced BW gain in TKO compared with TWT mice fed HFD diet was accompanied by improved glucose clearance an OGTT was performed. As expected, TWT-HFD mice had reduced glucose clearance compared to chow-fed mice. Of note, TKO-HFD mice had an overall glucose clearance comparable with that of chow-fed TKO and TWT mice, with a significantly lower blood glucose peak level after 15 and 30 min (Fig 3A and 3B), suggesting that lack of TIMP-1 protects against HFD-induced glucose intolerance. The protective effect against HFD-induced glucose intolerance in TKO mice was accompanied by a significant reduction in plasma glucose in non-fasted mice at the termination of the study (Fig 3C). However, no significant difference in plasma beta hydroxybutyrate (BHB) was observed in the two genotypes fed HFD (Fig 3D). It was anticipated that HFD induced insulin resistance in the TWT mice. Surprisingly, whole body insulin sensitivity, assessed by an i.p. insulin tolerance test (0.75 U/kg BW) was similar in all four groups (Fig 3E and 3F).

### TIMP-1 deficiency protects against HFD-induced hepatic inflammation and hepatic steatosis

**Fig 4:**
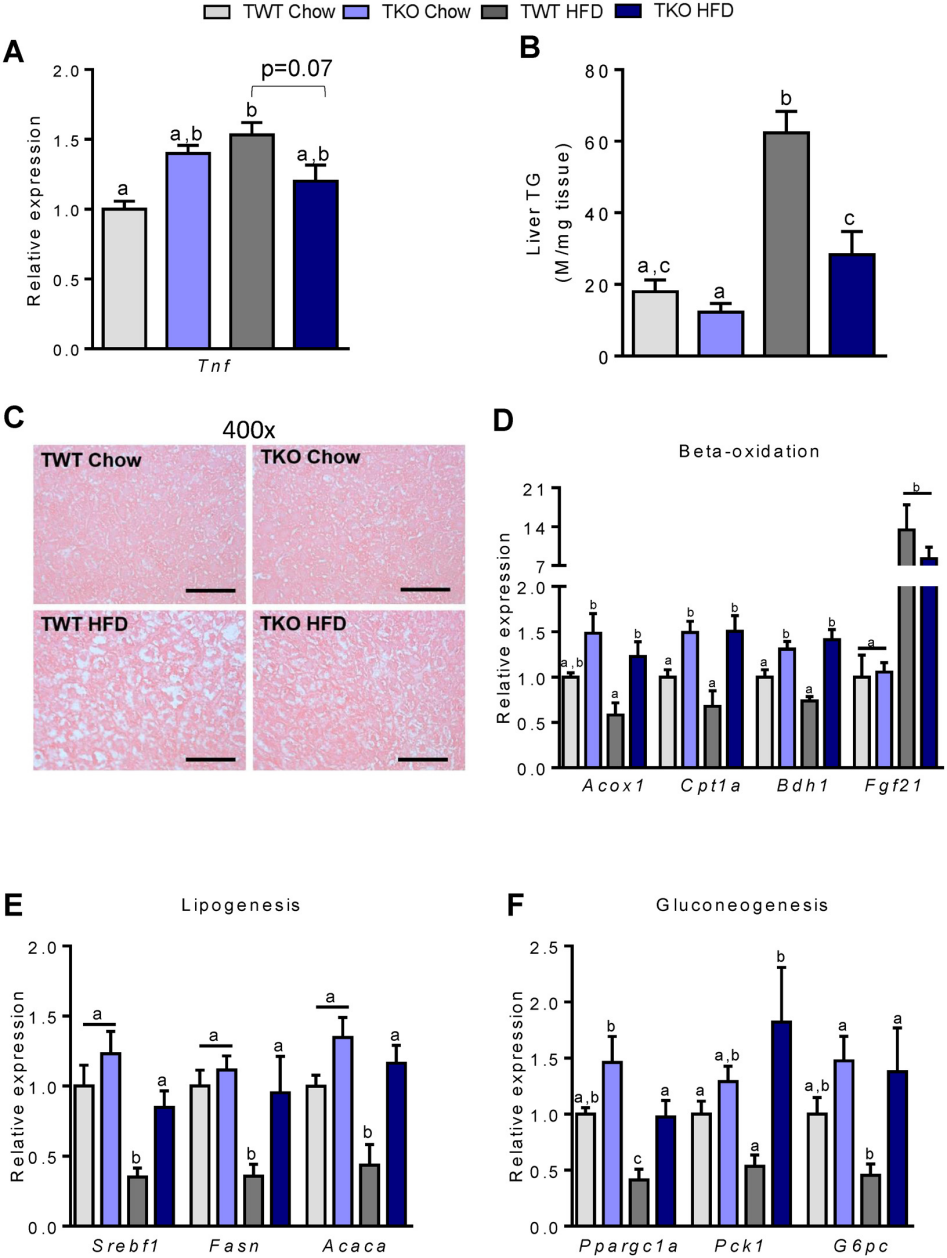
Hepatic fat metabolism in wild type (TWT) and *Timp1* knockout (TKO) mice fed chow or a high-fat diet (HFD). (A) Hepatic gene expression of *Tnf* (tumor necrosis factor α) measured by RT-qPCR. Data was normalized to 18S ribosomal RNA and presented relative to the expression in TWT Chow (n = 7–8). (B) Triglyceride content in liver (n = 9–11). (C) Liver sections stained with haematoxylin and eosin. A micrograph from one representative mouse in each group is shown, magnification x400, Scale bar = 200 μm. (D) Hepatic gene expression of acyl-CoA oxidase (*Acox1*), carnitine palmitoyl-CoA transferase-1a (*Cpt1a*), 3-hydroxybutyrate dehydrogenase, type 1 (*Bdh1*) and fibroblast growth factor 21 (*Fgf21*). (E) Hepatic gene expression of sterol regulatory element-binding protein-1c (*Srebf1*), fatty acid synthase (*Fasn*) and acyl-CoA carboxylase 1 (*Acaca*). (F) Hepatic gene expression of peroxisome proliferator-activated receptor γ coactivator 1α (*Ppargc1a*), phosphoenolpyruvate carboxykinase 1 (*Pck1*) and glucose-6-phosphatase, catalytic (*G6pc*). Gene expression was measured by RT-qPCR, normalized to 18S ribosomal RNA or general transcription factor II B (*gtf2b*) and presented relative to the expression in TWT Chow (n = 7–8). All RT-qPCR measurements were performed in non-fasted mice. Graphs show mean ± SEM, and different lowercase letters denote statistically different groups (p < 0.05).

Hepatic insulin resistance is associated with increased accumulation of lipids and expression of hepatic inflammatory markers [28]. Hepatic PGC1α has the ability to down-regulate several inflammatory cytokines, including tumor necrosis factor α (TNF-α), and is associated with hepatic insulin resistance [29,30]. We found a trend towards decreased *Tnf* expression (p = 0.07) in TKO compared to TWT mice fed HFD (Fig 4A). HFD increased hepatic fat accumulation in both TKO and TWT mice, but TKO-HFD mice accumulated significantly less hepatic fat than TWT-HFD mice (Fig 4B). Hepatic steatosis was confirmed in H&E stained liver sections showing large lipid droplets in TWT-HFD mice, whereas the appearance of TKO-HFD livers was more similar to that of chow fed animals (Fig 4C). However, although TWT mice developed signs of hepatic steatosis, staining of the hepatic sections did not stain positive with picrosirius red, indicating no deposition of collagen (S2 Fig). In addition, plasma levels of AST and ALT were normal (data not shown).

### TIMP-1 deficiency increases hepatic FFA metabolism in mice fed HFD

To elucidate possible molecular mechanisms by which TIMP-1-deficiency protects against HFD-induced hepatic lipid accumulation, the expression of genes controlling hepatic β-oxidation, lipogenesis and gluconeogenesis was analyzed. The expression levels of acyl-CoA oxidase (*Acox1*) and carnitine palmitoyl-CoA transferase-1a (*Cpt1a*) involved in peroxisomal and mitochondrial β-oxidation, respectively, and 3-hydroxybutyrate dehydrogenase, type 1 (*Bdh1*) were significantly higher in TKO-HFD mice compared to TWT-HFD and in TKO-chow compared to TWT-chow (Fig 4D). Expression level of *Fgf21* was increased by HFD, but the expression level was not influenced by TIMP-1 deletion. Unexpectedly, the expression of the lipogenic transcription factor sterol regulatory element-binding protein-1c (*Srebf1*) as well as the expression of fatty acid synthase (*Fasn*) and acyl-CoA carboxylase 1 (*Acaca*), the rate-limiting genes in lipogenesis, was higher in TKO-HFD compared to TWT-HFD mice (Fig 4E). Thus, genes involved in both fatty acid catabolism and anabolism were significantly higher expressed in livers from TKO-HFD than in TWT-HFD mice. This was accompanied by increased expression of *Ppargc1a*, phosphoenolpyruvate carboxykinase 1 (*Pck1*) and glucose-6-phosphatase, catalytic (*G6pc*), genes involved in gluconeogenesis (Fig 4F).

To investigate if TIMP-1 deletion influenced muscle metabolism, we measured triglyceride accumulation and the expression of genes involved in β-oxidation of fatty acids in the anterior tibialis muscle. We found no differences in lipid accumulation in the anterior tibialis muscle or in the expression of carnitine palmitoyl-CoA transferase-1b (*Cpt1b*) between the diets or genotypes. However, a significant induction in acyl-Coenzyme A dehydrogenase, medium chain (*Acadm)* was observed comparing chow and HFD fed TWT mice (S3 Fig).

### TIMP-1 deficiency protects against IFSD-induced glucose intolerance

**Fig 5:**
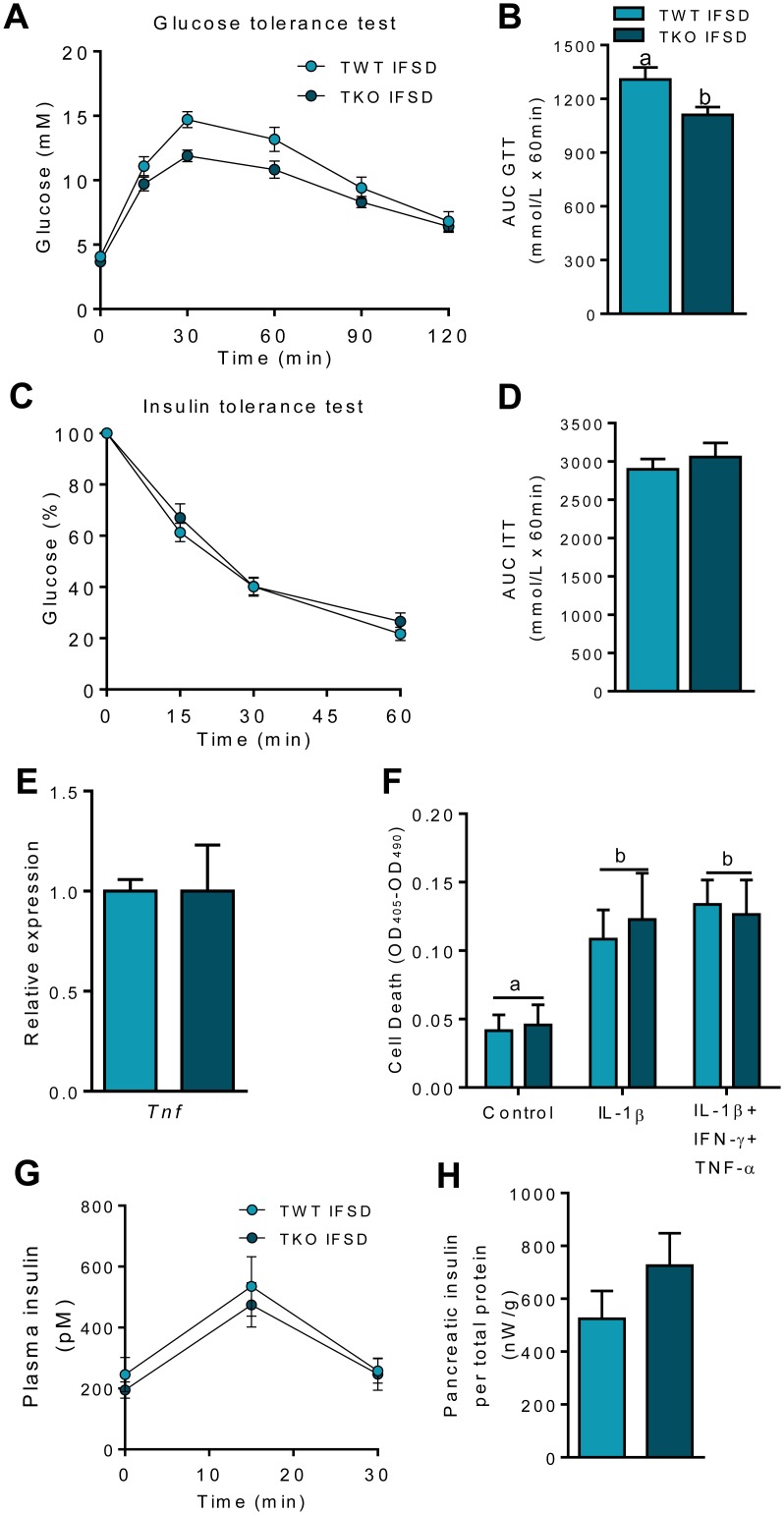
Glucose-, insulin tolerance test and beta cell function in wild type (TWT) and *Timp1* knockout (TKO) mice fed an intermediate fat and sucrose diet (IFSD). (A) Blood glucose (mM) levels after 23 weeks feeding before and after gavage of glucose (2 g/kg) (n = 8). (B) AUC values for glucose tolerance test (n = 8). (C) Blood glucose (mM) levels after 22 weeks feeding before and after i.p. injection of insulin (0.75 U/kg) (n = 8). (D) AUC values for insulin tolerance test (n = 8). (E) Hepatic gene expression of *Tnf* (tumor necrosis factor α) measured by RT-qPCR in random-fed mice. Data was normalized to 18S ribosomal RNA and presented relative to the expression in TWT IFSD (n = 7–8). All RT-qPCR measurements were performed in non-fasted mice. (F) Apoptosis in cell lysate from pancreatic islets from 12–14 week old male TWT or TKO mice. Islets were incubated with IL-1β (150 pg/ml) or IL-1β, IFN-γ (5 ng/ml) and TNF-α (10 ng/ml) for 24 hours and apoptosis was measured by DNA-histone complexes with Roche Cell Death Detection ELISA^PLUS^ (n = 6–8). (G) Plasma insulin levels after gavage of glucose (2 kg/g) in mice fed IFSD (n = 8). (H) Total pancreatic insulin after acid-ethanol insulin extraction from whole pancreas from mice fed IFSD (n = 4). Graphs show mean ± SEM, and different lowercase letters denote statistically different groups (p < 0.05).

To investigate whether the observed positive effects of TIMP-1 deficiency were linked to fat as energy source or to increased caloric intake *per se*, a second experiment was carried out where TWT and TKO mice were fed IFSD. The IFSD contained 25.1% fat and was supplemented with 43% sucrose to obtain a nearly isocaloric diet with the HFD (HFD: 5240 kcal/kg vs. IFSD: 4800 kcal/kg) (S1 Table). At termination, BW gain was similar in the two groups (S4A Fig). Due to higher feed intake the first six weeks of the study (data not shown), TKO-IFSD had a higher total feed intake than TWT-IFSD (S4B Fig). However, there was no difference in total energy efficiency (S4C Fig), mirrored by lack of differences in residual fecal calories, oxygen consumption and heat production (S4D–S4F Fig). Body composition and WAT mass were similar between genotypes (S4G and S4H Fig). Visual examination of eWAT and iWAT showed no morphological differences between the two genotypes (S5A Fig). After 23 weeks of feeding, TKO-IFSD had improved glucose tolerance compared to TWT-IFSD mice (Fig 5A and 5B). However, insulin sensitivity was not changed between the genotypes (Fig 5C and 5D). In addition, we found no differences in the hepatic expression of *Tnf* at the termination of the study (Fig 5E).

### TIMP-1 deficiency does not affect cytokine-induced beta cell apoptosis

To investigate whether the improved glucose clearance in TKO-IFSD compared with TWT-IFSD mice was due to the improved survival of beta cells in response to intra-islet low-grade inflammation usually observed in obesity, we exposed isolated TWT and TKO islets (S5B Fig) to IL-1β or a combination of IL-1β, IFN-γ, and TNF-α (cytomix) for 24 h and measured apoptosis (Fig 5F). Both IL-1β and cytomix induced apoptosis in TWT and TKO islets, but no differences could be observed between genotypes, despite TIMP-1 being expressed and upregulated in intact neonatal rat islets by cytokines (S5C and S5D Fig), suggesting TIMP-1 being dispensable in beta cell apoptosis.

### TIMP-1 deficiency does not affect glucose-stimulated insulin secretion

Plasma insulin concentrations during OGTT was quantified in order to further elucidate whether the improved glucose clearance in TKO-IFSD compared with TWT-IFSD was due to increased glucose-stimulated insulin secretion. However, no difference in glucose-stimulated insulin secretion was observed between TWT-IFSD and TKO-IFSD (Fig 5G). Furthermore, total islet insulin content was similar in TWT-IFSD and TKO-IFSD (Fig 5H), strengthening the notion that the role of TIMP-1 was unrelated to the response of the beta cell mass to low-grade islet inflammation.

### TIMP-1 deficiency protects against hepatic lipid accumulation in IFSD fed mice

**Fig 6:**
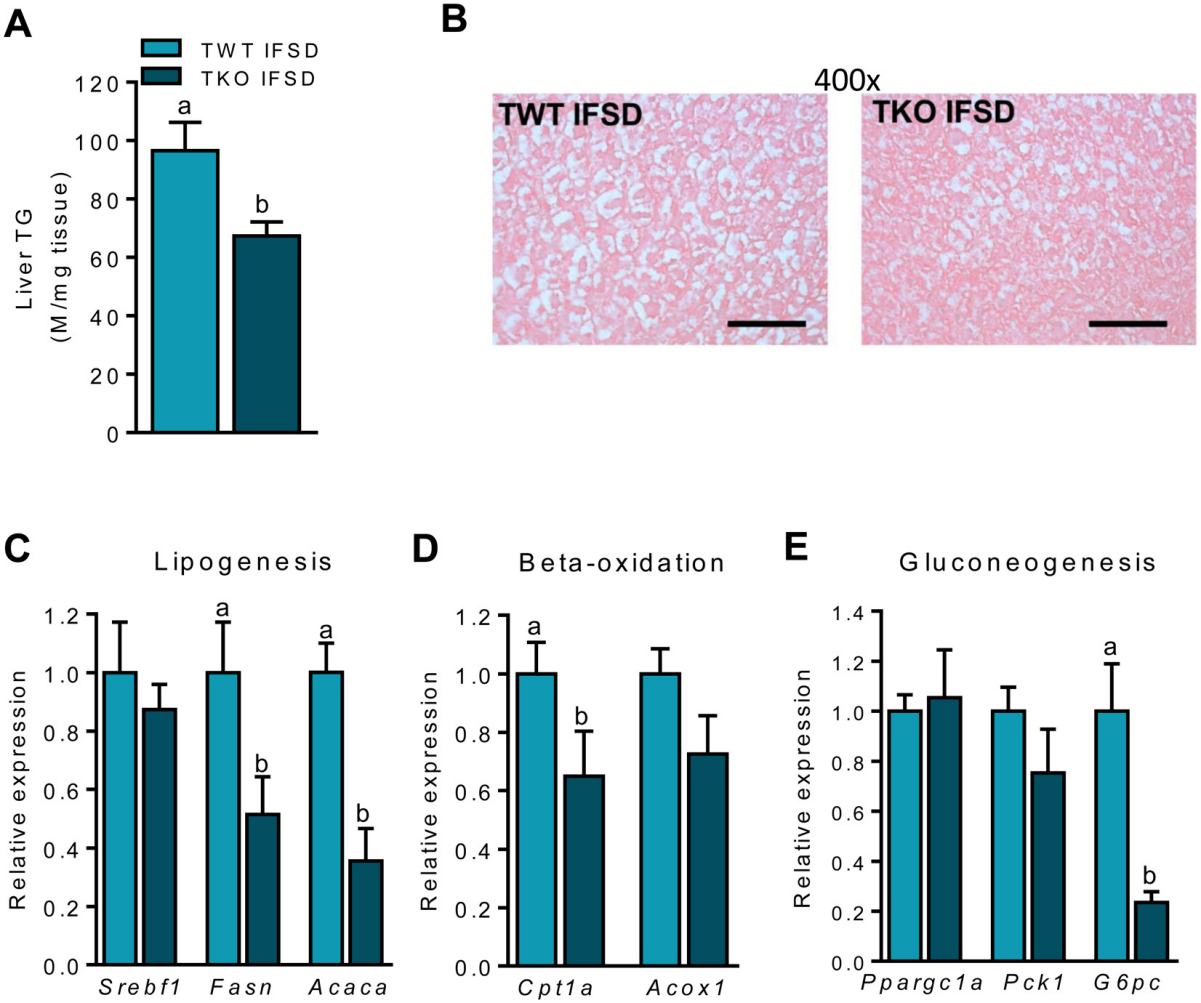
Hepatic fat metabolism in wild type (TWT) and *Timp1* knockout (TKO) mice fed an intermediate fat and sucrose diet (IFSD). (A) Triglyceride content in liver (n = 7–8). (B) Sections with liver tissues stained with haematoxylin and eosin. Micrographs from one representative mouse in each group are shown, magnification x400, scale bar = 200 μm. (C) Hepatic gene expression of sterol regulatory element-binding protein-1c (*Srebf1*), fatty acid synthase (*Fasn*) and acyl-CoA carboxylase 1 (*Acaca*). (D) Hepatic gene expression of carnitine palmitoyl-CoA transferase-1a (*Cpt1a*) and acyl-CoA oxidase (*Acox1*). (E) Hepatic gene expression of peroxisome proliferator-activated receptor γ coactivator 1α (*Ppargc1a*), phosphoenolpyruvate carboxykinase 1 (*Pck1*) and glucose-6-phosphatase, catalytic (*G6pc*). Gene expression was measured by RT-qPCR and normalized to 18S ribosomal RNA. Data is presented relative to the expression in TWT-IFSD (n = 7–8). All RT-qPCR measurements performed in random-fed mice. Graphs show mean ± SEM, and different lowercase letters denote statistically different groups (p < 0.05).

At the termination of the study, hepatic triglyceride accumulation was decreased in TKO-IFSD mice compared to TWT-IFSD (Fig 6A and 6B). To examine if the observed hepatic lipid accumulation in TWT-IFSD mice was associated with similar molecular changes as those observed in mice fed HFD, we measured the expression of genes controlling lipogenesis, β-oxidation and gluconeogenesis. The expression of *Fasn* and *Acaca* (involved in lipogenesis), *Cpt1a* (involved in β-oxidation) and *G6pc* (involved in gluconeogenesis) was reduced in TKO-IFSD mice compared to TWT-IFSD mice, while the expression of *Srebf1*, *Acox1*, *Ppargc1a* and *Pck1* was similar in the two genotypes (Fig 6C–6E). Taken together this suggests lower gluconeogenesis, lower lipogenesis and possibly also lower β-oxidation in the liver of TKO-IFSD compared to TWT-IFSD mice, potentially contributing to increased hepatic insulin sensitivity in TKO-IFSD mice.

Lipid accumulation in the anterior tibialis muscle and the expression of *Cpt1a* and *Acadm* was comparable between the two groups (S5E Fig).

## Discussion

Our results demonstrate that TIMP-1 deficiency partially protects against weight gain induced by HFD, but not by IFSD. TIMP-1 deficient mice were protected from HFD- and IFSD-induced development of hepatic steatosis and glucose intolerance, but TIMP-1 deficiency did not change peripheral insulin sensitivity or insulin secretion.

The mice used in this study were bred on a BALB/c background. BALB/c mice have an intermediate response to diet-induced diabetes [21]. The phenotypical changes might have been more pronounced if we had used an obesity-prone mouse strain, like the C57BL/6J mouse. However, we hypothesized that we could better mimic age-induced type 2 diabetes in the BALB/c background than in the obesity prone C57BL/6J background. In comparison with many other *in vivo* studies, the mice in our experiments were fed the diets for a relatively long time (26–29 weeks). We therefore believe that sustained exposure to a metabolic challenge, as done in this study, might more closely mimic the human situation than a short-term study with a very rapid and significant weight gain.

We confirmed the finding by Lijnen *et al*. [17] that TKO mice had a lower weight gain than TWT mice when fed a HFD. We also confirmed that TIMP-1 deficiency had no effect on BW or fat mass in chow-fed male mice [16,17]. However, we did not find a decreased size of adipocytes in WAT of TKO-HFD fed mice as reported by Lijnen *et al*. [17]. In contrast, Gerin *et al*. [16] found that female TKO mice on chow had increased BW compared to TWT mice, suggesting that the effect of TIMP-1 on BW could be gender-dependent. To our knowledge there are no studies assessing the effects of TIMP-1 in female mice fed obesogenic diets.

Our result demonstrating that TKO-HFD mice had lower feed efficiency compared to TWT-HFD mice is in agreement with earlier findings [17,20], but as reported by Meissburger *et al*. [20], the reduced feed efficiency was not accompanied by increased oxygen consumption. However, on HFD, but not on IFSD, we found that TIMP-1 deficiency was associated with increased energy content in feces, suggesting that TIMP-1 improves dietary fat uptake when dietary fat is in great excess. To our knowledge, the effects of TIMP-1 on dietary fat uptake have not previously been examined.

Both the HFD- and IFSD-induced reduction in glucose tolerance seen in TWT were improved in the TKO mice. However, the insulin tolerance test showed that peripheral insulin sensitivity was independent of genotype. The difference in glucose tolerance between the two genotypes could also not be explained by alterations in insulin secretion, i.e. there was no difference in insulin concentration in plasma after an oral glucose bolus and pancreatic insulin content analyzed *ex vivo* was similar in the two genotypes. Our finding that beta cell function was unrelated to TIMP-1 status is novel. Even though two previous studies [31,32] have shown that over-expression of human TIMP-1 or treatment with recombinant human TIMP-1 protects beta cells from inflammatory cell death, these findings are not necessarily in contrast to ours. Discrepancies might be due to the use of human recombinant TIMP-1 and/or locally highly increased TIMP-1 concentrations in these studies. Since we did not find improved peripheral insulin sensitivity or increased insulin secretion as an explanation for the higher glucose tolerance in TKO mice, we hypothesize that it might be due to enhanced hepatic insulin sensitivity. The i.p. total body insulin tolerance test is insensitive to discrete changes in hepatic insulin resistance [32]. However, the finding that hepatic expression of *Tnf* in TWT-HFD mice was elevated while unaffected in TKO-HFD mice compared to mice on chow is in keeping with the idea that insulin sensitivity may be reduced in TWT-HFD compared with TKO-HFD mice. Furthermore, we found that expression of *Ppargc1a* mRNA, recently linked to inhibition/modulation of obesity induced hepatic inflammation [29], was higher in TKO-HFD mice than in TWT-HFD mice, suggesting a mechanism for lack of hepatic inflammation in TKO-HFD mice. Of note, we found that TKO mice were protected or partially protected against the development of hepatic steatosis, often observed together with hepatic insulin resistance [33], further strengthening our hypothesis.

Another approach to assess the role of TIMP-1 on insulin sensitivity was taken by Meissburger *et al*. [20], who found that injections with murine TIMP-1 in mice accelerated insulin resistance and increased hepatic triacylglycerol accumulation, suggesting that TIMP-1 has a causal role in development of decreased glucose tolerance and hepatic steatosis. Meissburger *et al*. [20] suggest that impaired insulin sensitivity in this context was associated with an enlargement of adipocytes, leading to impaired uptake of glucose in adipose tissues as it is known that enlarged adipocytes become insulin resistant [34]. We observed no differences in peripheral insulin sensitivity between the two genotypes, but also, we did not observe differences in adipocyte sizes in the WAT in TWT and TKO mice.

Our findings suggest that TKO mice adapt better to an increased metabolic challenge than TWT mice. In TKO-HFD mice, increased dietary lipid content was associated with improved fat oxidation and increased gluconeogenesis as a response to the low sugar content of the diet compared to TWT-HFD mice. Additionally, TKO mice fed the IFSD with increased dietary sugar content had decreased expression of genes involved in gluconeogenesis, β-oxidation and lipogenesis compared to TWT-HFD mice.

Although metabolic studies of the effect of TIMP-1 have primarily focused on the role of TIMP-1 in obesity and WAT remodeling, the results obtained in this study and by Meissburger *et al*. [20], suggest that TIMP-1 plays an important role in hepatic insulin resistance and the development of steatosis. Binding of TIMP-1 to CD63 can lead to Akt activation [35]. Since hepatic overexpression of constitutively active Akt is known to induce hepatic steatosis [36], TIMP-1 may act through binding to CD63 and phosphorylation of Akt, thereby promoting hepatic steatosis. In a mouse model of hepatic inflammation and NASH, secretion of IL-1β induces hepatic stellate cell activation and TIMP-1 secretion, changing extracellular matrix expression, eventually leading to fibrosis [37]. The increased hepatic levels of *Ppargc1a* in TKO-HFD mice may counteract the effect of IL-1β through increased anti-inflammatory signaling [31], suggesting that TKO mice may also be protected against hepatic fibrosis. However, further studies are needed to elucidate the exact mechanisms of TIMP-1 in lipid metabolism and fibrosis in the liver.

In summary, this study shows that TIMP-1 differentially regulates hepatic metabolism dependent on fat and sucrose contents in the diet. We found TIMP-1 deficiency to be associated with altered hepatic metabolism, thereby contributing to improved hepatic function, decreased hepatic inflammation and decreased hepatic steatosis. TIMP-1 may represent a novel pharmacological target for improving hepatic insulin sensitivity in patients with the metabolic syndrome, NASH and Type 2 diabetes.

## Supporting Information

S1 Fig Body composition, gene expression of *Ccl2* and WAT morphology in wild type (TWT) and *Timp1* knockout (TKO) mice fed chow or a high-fat diet (HFD). (A) Body composition measured by scanning in week 26 (n = 10). (B) Gene expression of *Ccl2* (monocyte chemotactic protein-1) measured in eWAT and iWAT by RT-qPCR. Data was normalized to 18S ribosomal RNA and presented relative to the expression in TWT Chow (n = 7–8). All RT-qPCR measurements were performed in non-fasted mice. (C) H&E stained sections of eWAT and iWAT, scale bar = 100 μm. Average adipocyte diameter quantified in all experimental groups (n = 5). Graphs show mean ± SEM, and different lowercase letters denote statistically different groups (p < 0.05).
(TIF)

S2 Fig Picrosirius red stained sections of liver, eWAT and iWAT. One representative micrograph of each group is shown, scale bar = 100 μm (n = 5).
(TIF)

S3 Fig Triglyceride content and fat metabolism in anterior tibial muscle in wild type (TWT) and *Timp1* knockout (TKO) mice fed chow or a high-fat diet (HFD). Triglyceride content in the anterior tibialis muscle, and gene expression of *Cpt1b* (carnitine palmitoyl-CoA transferase-1a) and *Acadm* (medium-chain acyl-coenzyme A dehydrogenase) in anterior tibial muscle measured by RT-qPCR. Data was normalized to 18S ribosomal RNA and presented relative to the expression in TWT Chow (n = 7–8). All RT-qPCR measurements were performed in non-fasted mice. Graphs show mean ± SEM, and different lowercase letters denote statistically different groups (p < 0.05).
(TIF)

S4 Fig Body weight, feed intake, feed efficiency, indirect calorimetry and body composition in wild type (TWT) and *Timp1* knockout (TKO) mice fed an intermediate fat and sucrose diet (IFSD) for 29 weeks. (A) Total increase in body weight (n = 10). (B) Total energy intake (n = 10). (C) Energy efficiency at the end of the study measured as total body weight gain/total feed intake (n = 8). (D) Energy content in feces measured by calorimetry (n = 8). (E) Oxygen consumption and (F) heat production, both measured over a 24 h period with indirect calorimetry in week 20 (n = 8). (G) Body composition measured by scanning (n = 10). (H) Weight of total WAT, epididymal white adipose tissue (eWAT), inguinal white adipose tissue (iWAT) and perirenal white adipose tissue (pWAT) at the end of the study (n = 7–8). Graphs show mean ± SEM, and different lowercase letters denote statistically different groups (p < 0.05).
(TIF)

S5 Fig WAT morphology, beta-cell function and fat metabolism in anterior tibial muscle in wild type (TWT) and *Timp1* knockout (TKO) mice fed an intermediate fat and sucrose diet (IFSD) for 29 weeks. (A) H&E stained sections of eWAT and iWAT, scale bar = 100 μm. Average adipocyte diameter quantified in both experimental groups (n = 5). (B) TIMP-1 gene expression in isolated TWT and TKO islets. (C) Representative Western blot and (D) Quantification relative to tubulin of TIMP-1 protein expression in neonatal rat islets (n = 2–4). Islets were exposed to a combination of IL-1β (150 pg/ml) and IFN-γ (5 ng/ml) for 18 or 24 hours, or IL-1β (150 pg/ml), IFN-γ (5 ng/ml) and TNF-α (10 ng/ml) for 24 hours. (E) Triglyceride content in the anterior tibialis muscle, and gene expression of *Cpt1b* (carnitine palmitoyl-CoA transferase-1a) and *Acadm* (medium-chain acyl-coenzyme A dehydrogenase) in anterior tibial muscle measured by RT-qPCR. Data was normalized to 18S ribosomal RNA and presented relative to the expression in TWT Chow (n = 7–8). All RT-qPCR measurements were performed in randomly selected fed mice. Graphs show mean ± SEM, and different lowercase letters denote statistically different groups (p < 0.05).
(TIF)

S1 Table Dietary macronutrient composition of the experimental diets. (TIF)

S2 Table Primer sequences. (TIF)
