# Supplementary figures and images for: Tissue Inhibitor Of Matrix Metalloproteinase-1 Is Required for High-Fat Diet-Induced Glucose Intolerance and Hepatic Steatosis in Mice
Source: PLoS One. 2015 Jul 13;10(7):e0132910. doi: 10.1371/journal.pone.0132910 (PMC4500465; doi:10.1371/journal.pone.0132910)

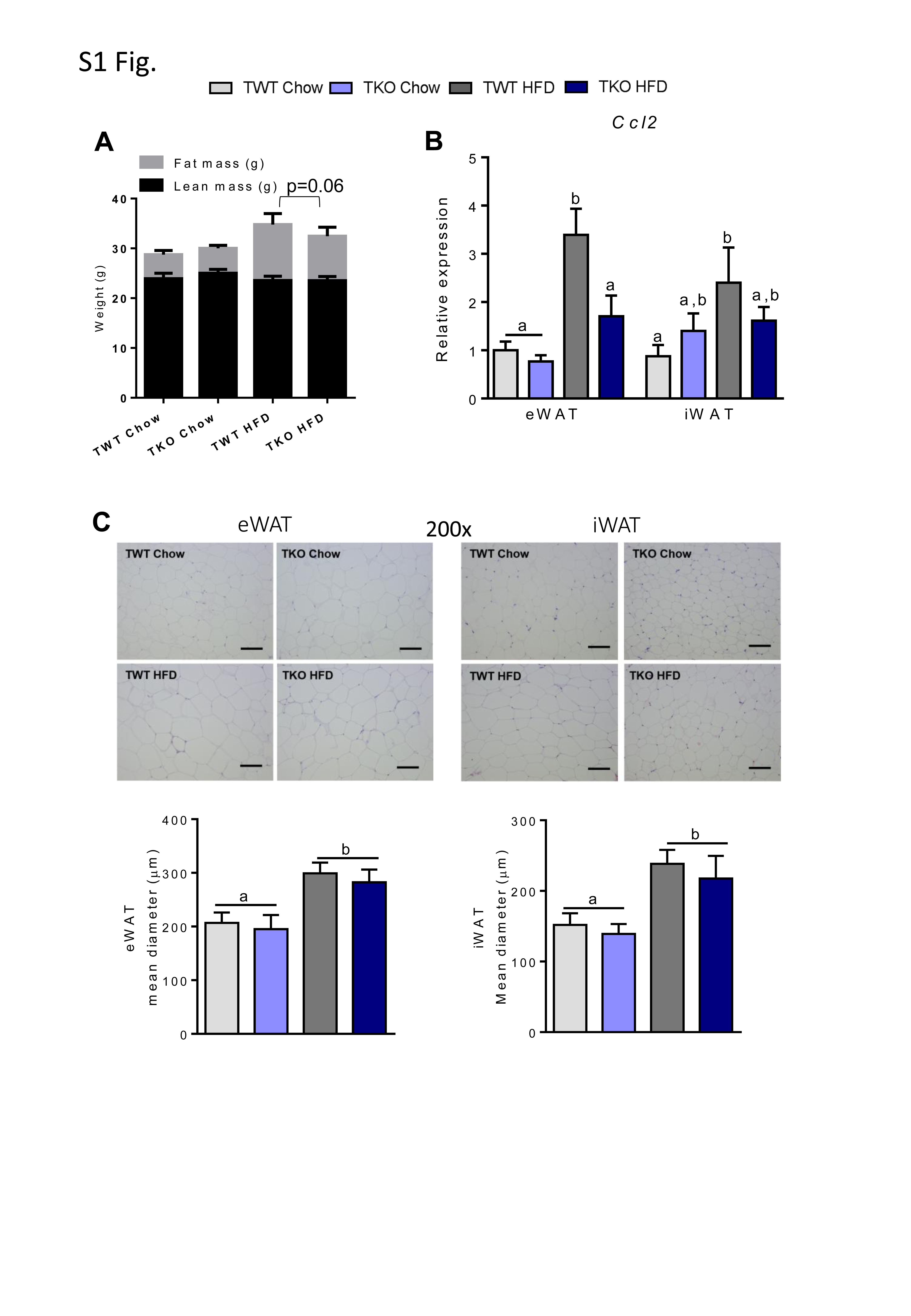

Supplement: S1 Fig — (A) Body composition measured by scanning in week 26 (n = 10). (B) Gene expression of Ccl2 (monocyte chemotactic protein-1) measured in eWAT and iWAT by RT-qPCR. Data was normalized to 18S ribosomal RNA and presented relative to the expression in TWT Chow (n = 7–8). All RT-qPCR measurements were performed in non-fasted mice. (C) H&E stained sections of eWAT and iWAT, scale bar = 100 μm. Average adipocyte diameter quantified in all experimental groups (n = 5). Graphs show mean ± SEM, and different lowercase letters denote statistically different groups (p < 0.05). (TIF) [file pone.0132910.s001.tif]

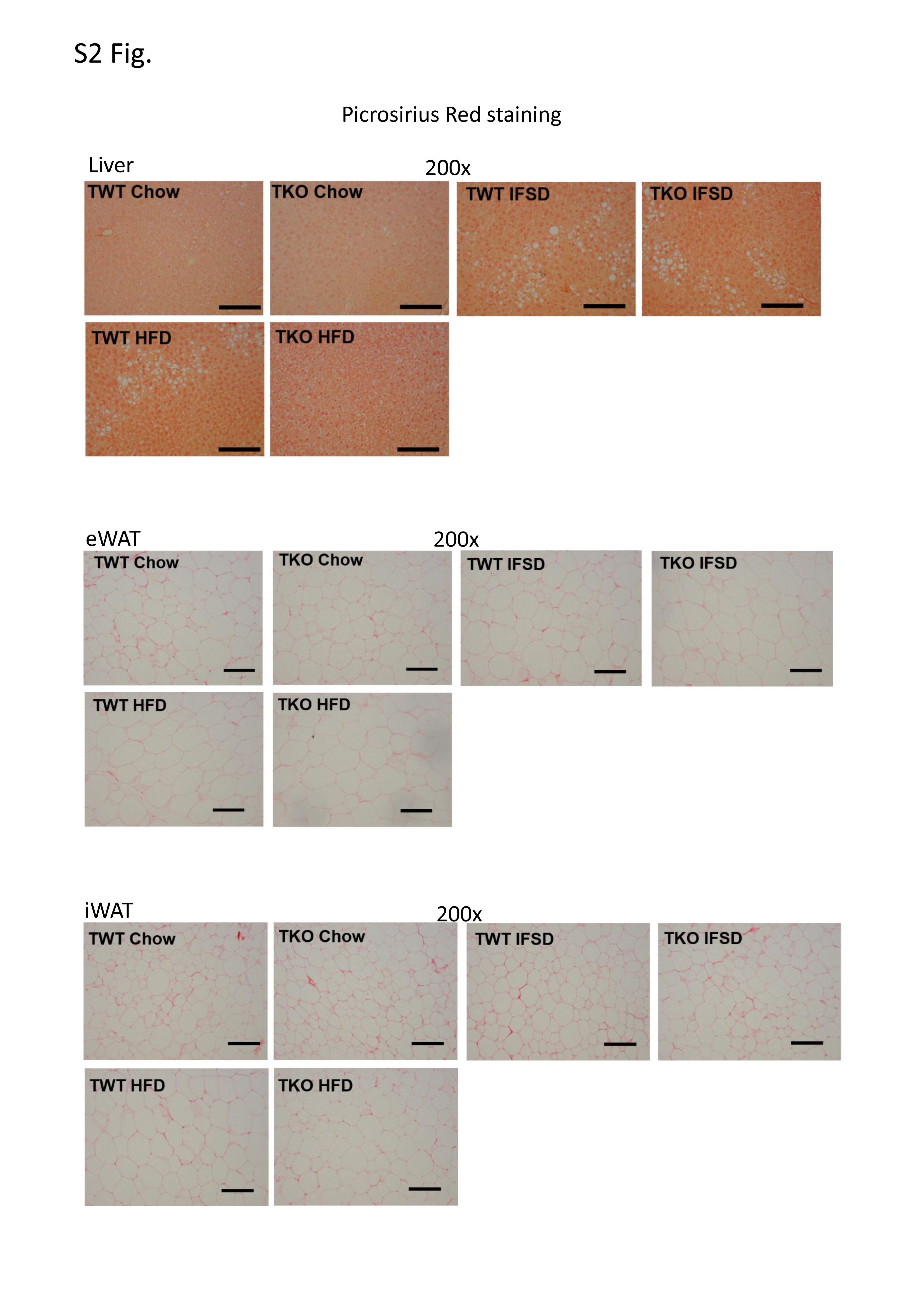

Supplement: S2 Fig — One representative micrograph of each group is shown, scale bar = 100 μm (n = 5). (TIF) [file pone.0132910.s002.tif]

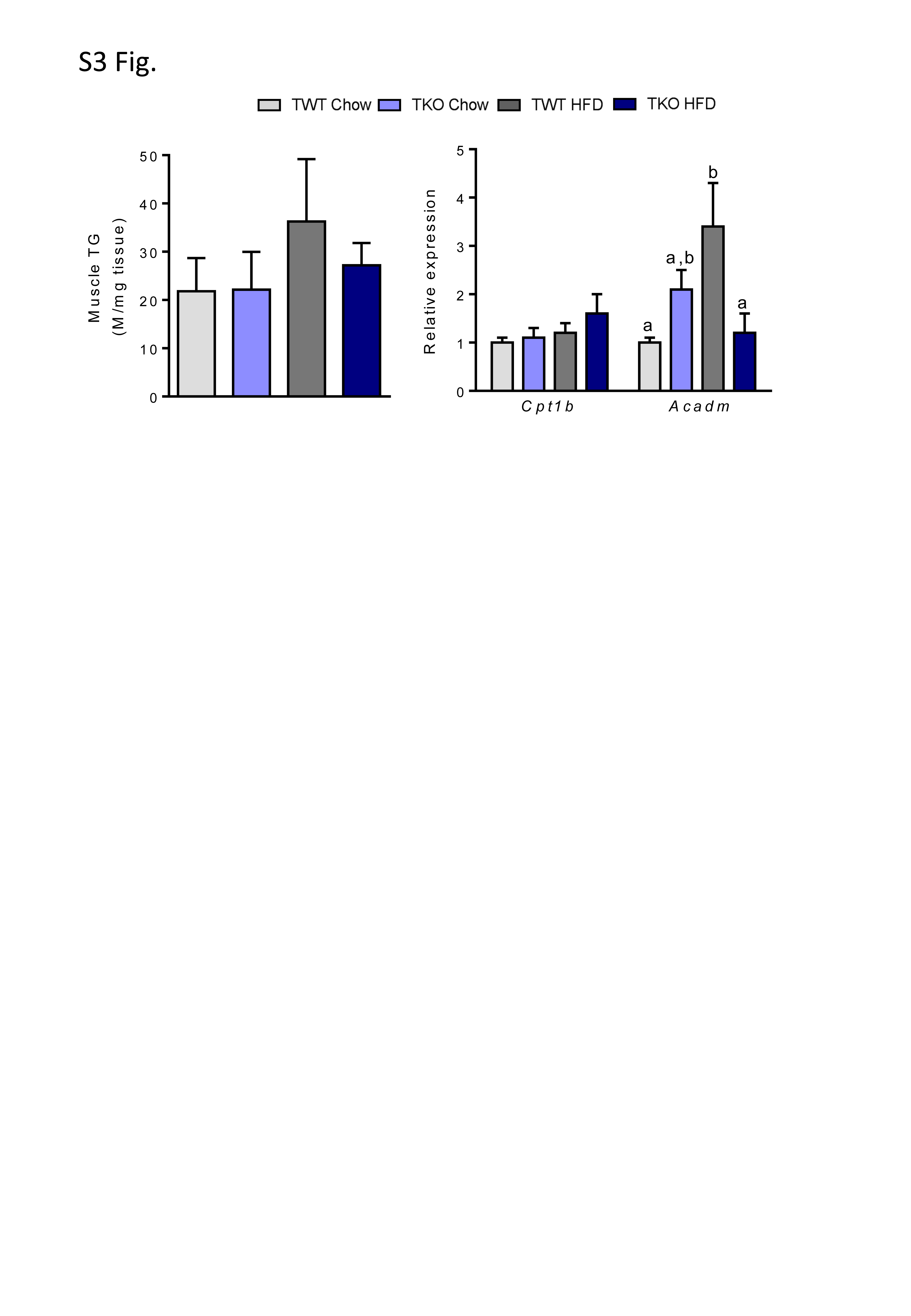

Supplement: S3 Fig — Triglyceride content in the anterior tibialis muscle, and gene expression of Cpt1b (carnitine palmitoyl-CoA transferase-1a) and Acadm (medium-chain acyl-coenzyme A dehydrogenase) in anterior tibial muscle measured by RT-qPCR. Data was normalized to 18S ribosomal RNA and presented relative to the expression in TWT Chow (n = 7–8). All RT-qPCR measurements were performed in non-fasted mice. Graphs show mean ± SEM, and different lowercase letters denote statistically different groups (p < 0.05). (TIF) [file pone.0132910.s003.tif]

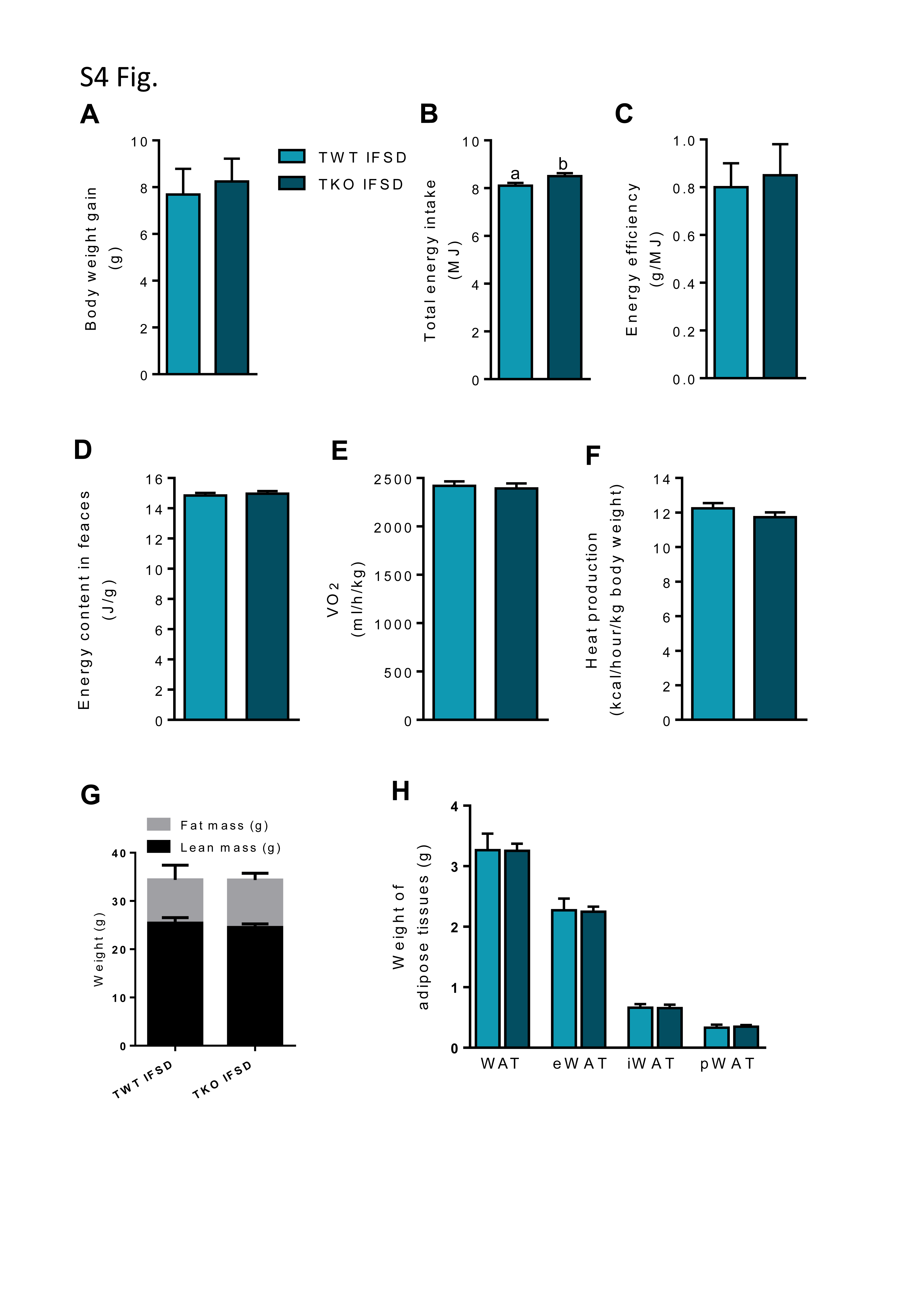

Supplement: S4 Fig — (A) Total increase in body weight (n = 10). (B) Total energy intake (n = 10). (C) Energy efficiency at the end of the study measured as total body weight gain/total feed intake (n = 8). (D) Energy content in feces measured by calorimetry (n = 8). (E) Oxygen consumption and (F) heat production, both measured over a 24 h period with indirect calorimetry in week 20 (n = 8). (G) Body composition measured by scanning (n = 10). (H) Weight of total WAT, epididymal white adipose tissue (eWAT), inguinal white adipose tissue (iWAT) and perirenal white adipose tissue (pWAT) at the end of the study (n = 7–8). Graphs show mean ± SEM, and different lowercase letters denote statistically different groups (p < 0.05). (TIF) [file pone.0132910.s004.tif]

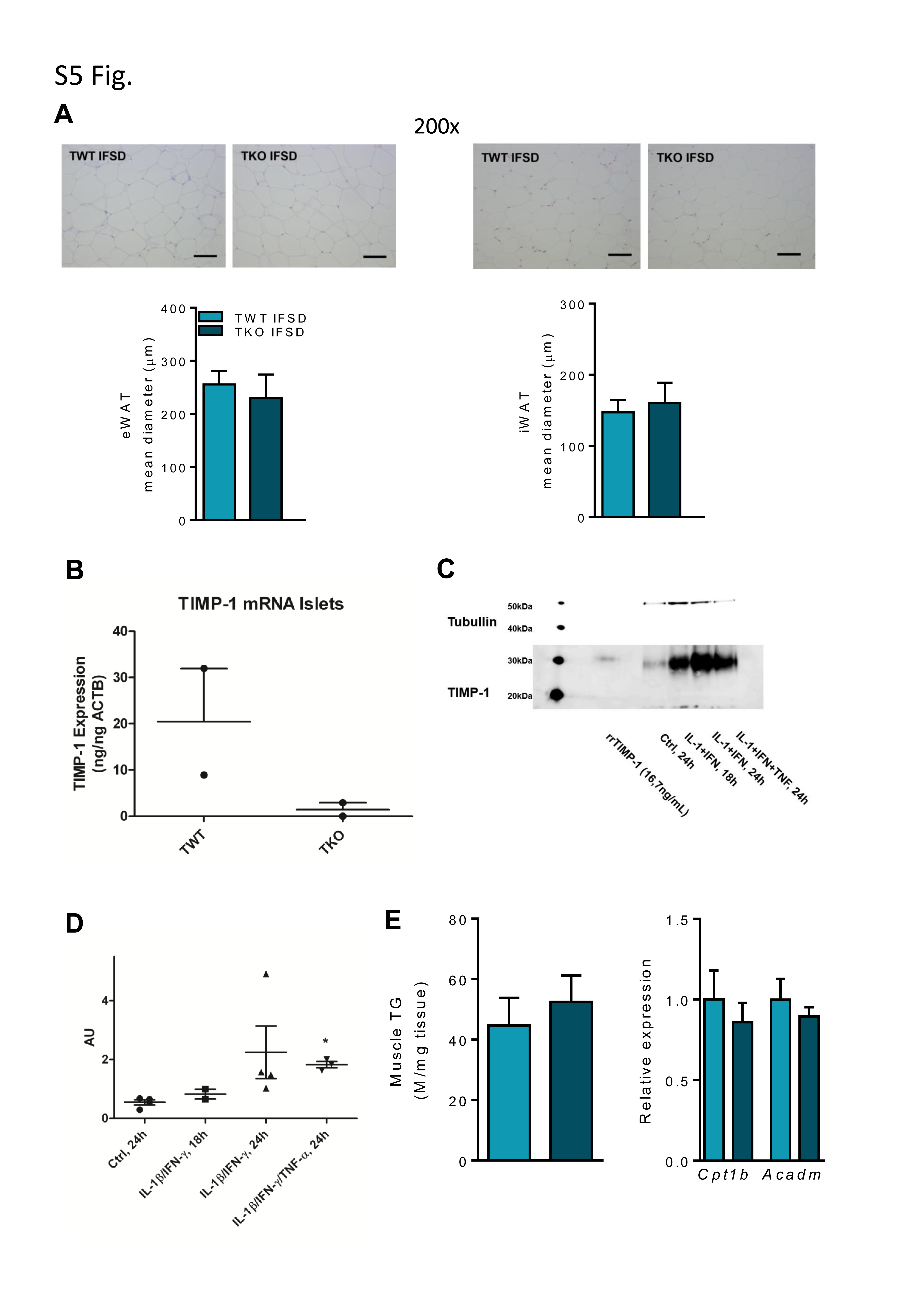

Supplement: S5 Fig — (A) H&E stained sections of eWAT and iWAT, scale bar = 100 μm. Average adipocyte diameter quantified in both experimental groups (n = 5). (B) TIMP-1 gene expression in isolated TWT and TKO islets. (C) Representative Western blot and (D) Quantification relative to tubulin of TIMP-1 protein expression in neonatal rat islets (n = 2–4). Islets were exposed to a combination of IL-1β (150 pg/ml) and IFN-γ (5 ng/ml) for 18 or 24 hours, or IL-1β (150 pg/ml), IFN-γ (5 ng/ml) and TNF-α (10 ng/ml) for 24 hours. (E) Triglyceride content in the anterior tibialis muscle, and gene expression of Cpt1b (carnitine palmitoyl-CoA transferase-1a) and Acadm (medium-chain acyl-coenzyme A dehydrogenase) in anterior tibial muscle measured by RT-qPCR. Data was normalized to 18S ribosomal RNA and presented relative to the expression in TWT Chow (n = 7–8). All RT-qPCR measurements were performed in randomly selected fed mice. Graphs show mean ± SEM, and different lowercase letters denote statistically different groups (p < 0.05). (TIF) [file pone.0132910.s005.tif]

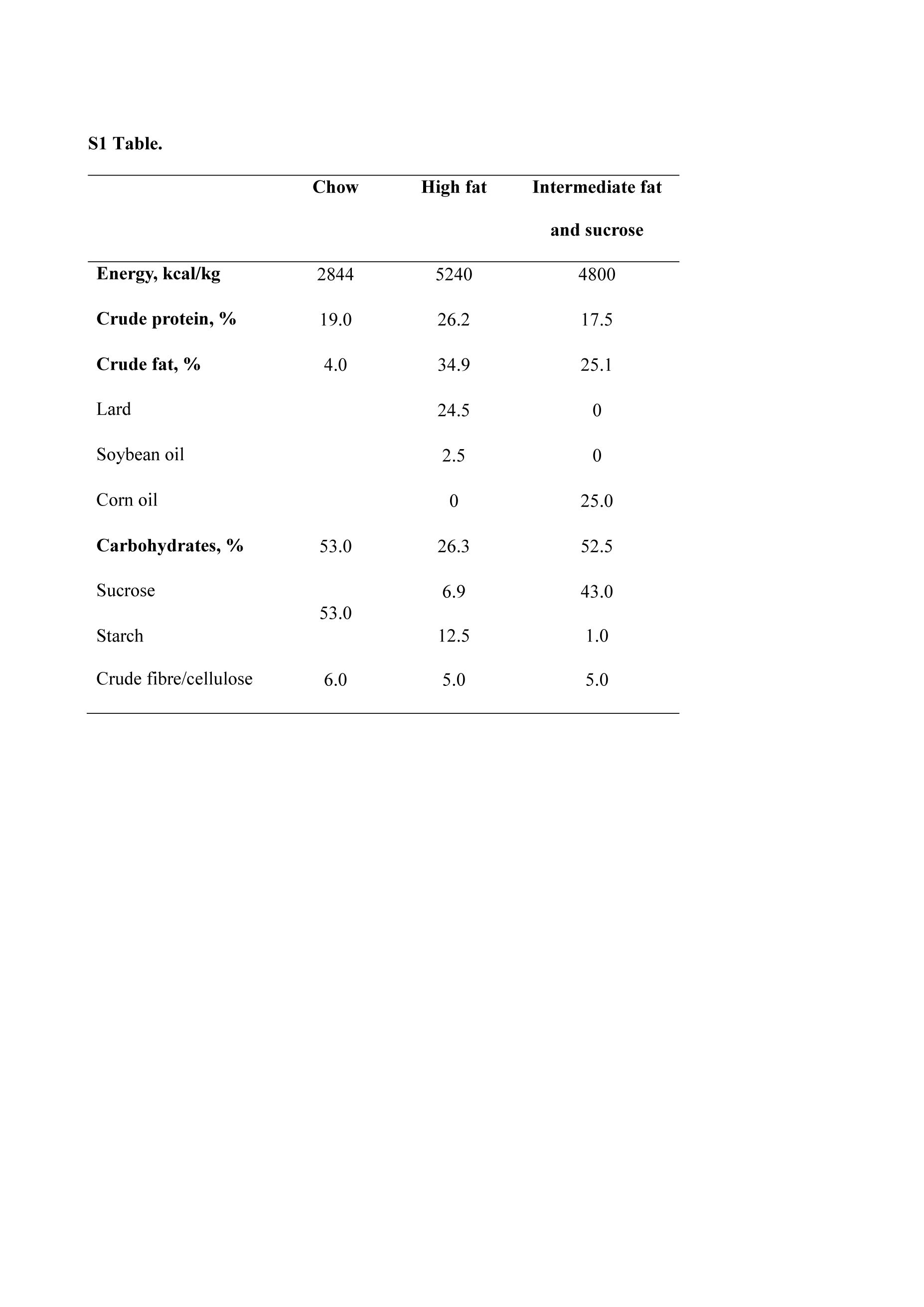

Supplement: S1 Table — (TIF) [file pone.0132910.s006.tif]

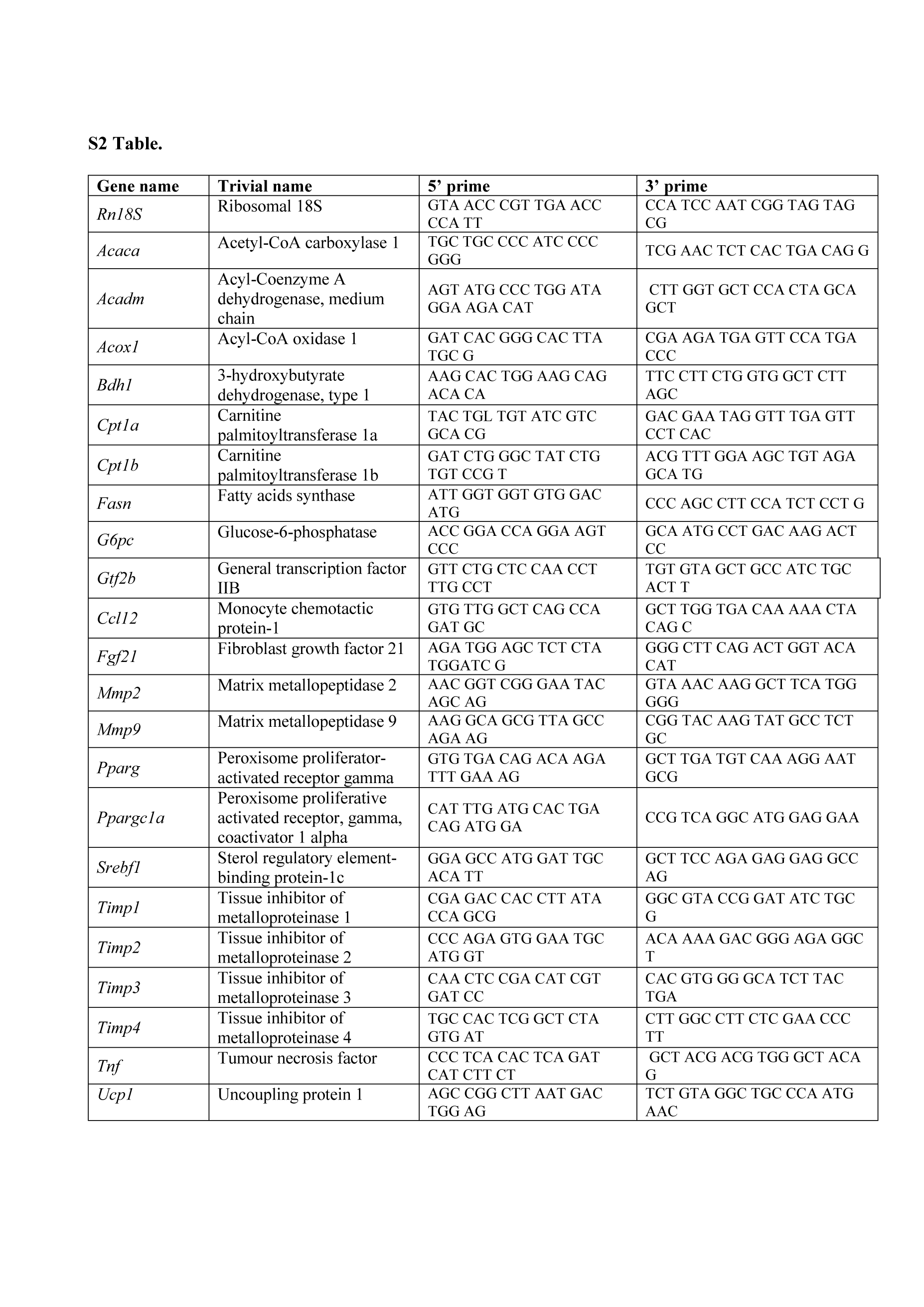

Supplement: S2 Table — (TIF) [file pone.0132910.s007.tif]
